# Impact of person-centred care training and person-centred activities on quality of life, agitation, and antipsychotic use in people with dementia living in nursing homes: A cluster-randomised controlled trial

**DOI:** 10.1371/journal.pmed.1002500

**Published:** 2018-02-06

**Authors:** Clive Ballard, Anne Corbett, Martin Orrell, Gareth Williams, Esme Moniz-Cook, Renee Romeo, Bob Woods, Lucy Garrod, Ingelin Testad, Barbara Woodward-Carlton, Jennifer Wenborn, Martin Knapp, Jane Fossey

**Affiliations:** 1 Exeter University Medical School, Exeter University, Exeter, United Kingdom; 2 Institute of Mental Health, University of Nottingham, Nottingham, United Kingdom; 3 Division of Psychiatry and Applied Psychology, Institute of Mental Health, University of Nottingham, Nottingham, United Kingdom; 4 Wolfson Centre for Age-Related Diseases, King’s College London, London, United Kingdom; 5 Faculty of Health and Social Sciences, University of Hull, Hull, United Kingdom; 6 Oxford Health NHS Foundation Trust, Oxford, United Kingdom; 7 Dementia Services Development Centre Wales, Bangor University, Bangor, United Kingdom; 8 Centre for Age-related Medicine (SESAM), Helse Stavanger University Hospital, Stavanger, Norway; 9 Alzheimer’s Society, London, United Kingdom; 10 Division of Psychiatry, University College London, London, United Kingdom; 11 London School of Economics, London, United Kingdom; University of California San Francisco Memory and Aging Center, UNITED STATES

## Abstract

**Background:**

Agitation is a common, challenging symptom affecting large numbers of people with dementia and impacting on quality of life (QoL). There is an urgent need for evidence-based, cost-effective psychosocial interventions to improve these outcomes, particularly in the absence of safe, effective pharmacological therapies. This study aimed to evaluate the efficacy of a person-centred care and psychosocial intervention incorporating an antipsychotic review, WHELD, on QoL, agitation, and antipsychotic use in people with dementia living in nursing homes, and to determine its cost.

**Methods and findings:**

This was a randomised controlled cluster trial conducted between 1 January 2013 and 30 September 2015 that compared the WHELD intervention with treatment as usual (TAU) in people with dementia living in 69 UK nursing homes, using an intention to treat analysis. All nursing homes allocated to the intervention received staff training in person-centred care and social interaction and education regarding antipsychotic medications (antipsychotic review), followed by ongoing delivery through a care staff champion model. The primary outcome measure was QoL (DEMQOL-Proxy). Secondary outcomes were agitation (Cohen-Mansfield Agitation Inventory [CMAI]), neuropsychiatric symptoms (Neuropsychiatric Inventory–Nursing Home Version [NPI-NH]), antipsychotic use, global deterioration (Clinical Dementia Rating), mood (Cornell Scale for Depression in Dementia), unmet needs (Camberwell Assessment of Need for the Elderly), mortality, quality of interactions (Quality of Interactions Scale [QUIS]), pain (Abbey Pain Scale), and cost. Costs were calculated using cost function figures compared with usual costs. In all, 847 people were randomised to WHELD or TAU, of whom 553 completed the 9-month randomised controlled trial. The intervention conferred a statistically significant improvement in QoL (DEMQOL-Proxy *Z* score 2.82, *p =* 0.0042; mean difference 2.54, SEM 0.88; 95% CI 0.81, 4.28; Cohen’s *D* effect size 0.24). There were also statistically significant benefits in agitation (CMAI *Z* score 2.68, *p =* 0.0076; mean difference 4.27, SEM 1.59; 95% CI −7.39, −1.15; Cohen’s *D* 0.23) and overall neuropsychiatric symptoms (NPI-NH *Z* score 3.52, *p* < 0.001; mean difference 4.55, SEM 1.28; 95% CI −7.07,−2.02; Cohen’s *D* 0.30). Benefits were greatest in people with moderately severe dementia. There was a statistically significant benefit in positive care interactions as measured by QUIS (19.7% increase, SEM 8.94; 95% CI 2.12, 37.16, *p =* 0.03; Cohen’s *D* 0.55). There were no statistically significant differences between WHELD and TAU for the other outcomes. A sensitivity analysis using a pre-specified imputation model confirmed statistically significant benefits in DEMQOL-Proxy, CMAI, and NPI-NH outcomes with the WHELD intervention. Antipsychotic drug use was at a low stable level in both treatment groups, and the intervention did not reduce use. The WHELD intervention reduced cost compared to TAU, and the benefits achieved were therefore associated with a cost saving. The main limitation was that antipsychotic review was based on augmenting processes within care homes to trigger medical review and did not in this study involve proactive primary care education. An additional limitation was the inherent challenge of assessing QoL in this patient group.

**Conclusions:**

These findings suggest that the WHELD intervention confers benefits in terms of QoL, agitation, and neuropsychiatric symptoms, albeit with relatively small effect sizes, as well as cost saving in a model that can readily be implemented in nursing homes. Future work should consider how to facilitate sustainability of the intervention in this setting.

**Trial registration:**

ISRCTN Registry ISRCTN62237498

## Abstract 2

In a cluster-randomized controlled trial, Clive Ballard and colleagues test whether a program combining staff training, social interaction, and guidance on use of antipsychotic medications improves quality of life and symptoms among people with dementia living in UK care homes.

## Author summary

**Why was this study done?:**

- People with dementia living in care homes often experience agitation and other symptoms that are difficult to treat and distressing for the individual.

**What did the researchers do and find?:**

- We tested the WHELD programme, which combined staff training, social interaction, and guidance on use of antipsychotic medications, in 69 UK care homes in a 9-month clinical trial.
- We showed that care homes receiving the WHELD programme saw improvements in quality of life as well as other important symptoms including agitation, behaviour, and pain in people with dementia.
- The WHELD programme was also shown to be cost-effective.

**What do these findings mean?:**

- The findings show that the WHELD approach is beneficial for people with dementia living in care homes.
- WHELD could be provided in an affordable way to improve the lives of these individuals, who often do not receive the care they need.

## Introduction

There are 46.8 million people with dementia worldwide, many of whom reside in nursing homes. In the UK one-third of people with dementia live in care homes [1], and in the US 64% of people receiving Medicare in nursing homes have dementia [2], demonstrating the international impact of the condition. The majority of these individuals have moderate or severe dementia and have highly complex care needs resulting from a combination of cognitive, functional, and communication impairments; neuropsychiatric symptoms; and medical comorbidity, all of which combine to impact on quality of life (QoL). Interventions to promote QoL in dementia are limited in the literature, and few trials have examined impacts on this important outcome. Despite the close link of QoL to agitation and other neuropsychiatric symptoms, risk of falls, worsening cognition, and mortality, none of the 18 randomised controlled trials (RCTs) found in 2 systematic reviews of antipsychotic medication use in individuals with dementia measured QoL as an outcome [3,4]. There is considerable potential for non-drug approaches to address major drivers of QoL, and a recent systematic review particularly highlighted the benefit conferred by social interaction and pleasant activities in decreasing both agitation and antipsychotic use [5]. To date, interventions to promote person-centred care (PCC) have not achieved a significant improvement in QoL for people with dementia [6–8]. The exception is a recently published intensive proof-of-concept study that confirmed the added benefits of combining PCC training for care staff, antipsychotic review, and social interaction—the WHELD intervention—and demonstrated significant benefits in QoL, as well as a significant reduction in antipsychotic use [9].

Neuropsychiatric symptoms affect 90% of people with dementia at some point during the course of their condition [10]. Agitation, frequently including aggression, is particularly common amongst those with moderate to severe dementia living in nursing homes, where the cross-sectional prevalence of these symptoms exceeds 50% [11]. Agitation is associated with increased distress in residents and an increased burden for family and professional caregivers [4] and is one of the most challenging symptoms for clinical management. Importantly, agitation is closely associated with reduced QoL in people with dementia. There is evidence to support modest benefits of antipsychotic treatment for some symptoms of agitation, particularly risperidone, olanzapine, and aripiprazole for the short-term management of severe aggression. The benefits for other symptoms of agitation and with longer term treatment are less clear [12–15]. Moreover, antipsychotics are associated with severe safety concerns including increased cognitive decline, stroke, and death, particularly when used in the long term [13,15–17]. Best practice guidance emphasises the importance of frequent monitoring and judicious prescribing in order to reduce these risks but also to ensure identification of situations where antipsychotic use is warranted [18,19]. Recent studies also highlight emerging pharmacological alternatives to antipsychotic medications. The CitAD trial examined treatment with citalopram for 9 weeks in 186 people with Alzheimer disease and reported a significant reduction in agitation (odds ratio 2.13, *p =* 0.01) and caregiver distress [20], and a trial of dextromethorphan-quinidine in 194 people with Alzheimer disease reported a clinically relevant benefit for agitation (ordinary least squares *Z* statistic −3.95, *p* < 0.001) over a 10-week treatment period [21]. Whilst this emerging evidence base is promising, there were safety concerns with citalopram, and both the citalopram and dextromethorphan-quinidine studies only evaluated relatively short-term therapy (9–10 weeks). There is also currently a lack of evidence supporting sustained benefit for any current pharmacological treatment for agitation.

Livingston and colleagues [22] in a comprehensive systematic review examined the benefits of a range of sensory, psychological, and behavioural interventions in the treatment of agitation. The authors identified 160 clinical trials and reported promising indications of benefit across a range of interventions. A parallel systematic review concentrating specifically on psychological and behavioural interventions identified 40 clinical trials in people with dementia, and, together with other key studies, highlights the potential value of enjoyable activities as a successful treatment approach for agitation [5,23,24]. A more specific systematic review and meta-analysis concentrating on parallel group clinical trials of dementia-related PCC training identified 5 trials. A meta-analysis of these studies demonstrated significant benefits in the treatment of agitation and in achieving reductions in the use of antipsychotic medications. However, no significant benefits in improving QoL were achieved [8]. This literature highlights the growing evidence base to support the value of PCC and non-pharmacological interventions for the management of agitation and reduction of antipsychotic use for people with dementia in nursing homes, which was further augmented by a recently published factorial study of the WHELD intervention [9].

Cost is a major consideration in the development and implementation of interventions in nursing homes [5,8,9]. None of the evidence-based interventions to promote PCC have been widely adopted in clinical and care practice, and this is likely due in part to a lack of robust evidence regarding the cost profile of these approaches. A potentially cost-effective, practical means of overcoming this issue is to deliver interventions through a champion model, enabling care staff to take ownership for ongoing implementation in the care home, with more limited supervision from external therapists.

The goal of this RCT was to evaluate the impact of the WHELD intervention on QoL, agitation, neuropsychiatric symptoms, antipsychotic use, global deterioration, mood, unmet needs, mortality, quality of interactions, pain, and cost in comparison to treatment as usual (TAU).

## Methods

### Study design

This study was a 9-month cluster-randomised controlled 2-arm trial conducted in 69 UK nursing homes between 1 January 2013 and 30 September 2015. There were 3 recruiting hubs based in South London, North London, and Buckinghamshire. Each cluster was randomised to receive either the WHELD intervention or TAU for 9 months. This research was reviewed and approved by the Oxford C National Research Ethics Committee (Ref: 13/SC/0281). This study is registered with the ISRCTN Registry (Ref: ISRCTN62237498). The full protocol is available in the published protocol paper [25].

### Eligibility criteria

Eligible nursing homes had at least 60% of residents with dementia. Nursing homes were excluded if they were receiving special support from their local authority or if they failed to meet the 5 Care Quality Commission care home quality standards. Within each participating nursing home, all residents were considered potentially eligible for inclusion if they met criteria for dementia (defined as a score 1 or greater on the Clinical Dementia Rating [CDR] [26], operationalized to require a minimum level of cognitive, functional, and neuropsychiatric features).

### Interventions

The WHELD intervention consisted of a combination of elements taken from the interventions evaluated in a previous proof-of-concept study [9]. The intervention focused on training in PCC for care staff and on promoting tailored person-centred activities and social interactions. The intervention also involved the development of a system for triggering appropriate review of antipsychotic medications by the prescribing physician attached to each home.

Training for staff was provided by a research therapist. Two lead care staff members (WHELD champions) were nominated in each care home. These individuals received additional training over a period of 4 months (1 training day per month) with further coaching, supervision, and regular review with the therapist over the 9-month period. The WHELD champions were responsible for the delivery and dissemination of the intervention in each care home. In addition, prescribing physicians were provided with educational materials about the intervention. The control group received TAU. The WHELD intervention is described in more detail in Box 1 and S1 Table.

**Box 1. Details of WHELD intervention**

**Orientation phase**

**Duration:** 2 whole days or 4 half days in each home over 1 month.

**Delivered by:** 1 full-time WHELD therapist for each 9 care homes.

**Participants:** Care home managers, staff teams, WHELD champions, and residents.

**Aim:** The WHELD therapist met with residents and staff to introduce the project and provide information, meet nominated WHELD champions, understand staff hopes and concerns, and review the layout and facilities of the care home where the intervention would take place.

**Intervention delivery phase**

**Duration:** 8 months (months 2–9).

**(1) Months 2–5:** Training delivered to WHELD champions off-site from the care home where they work for 1 day (6 hours) per month for each care home.

**Delivered by:** WHELD therapist.

**Participants:** WHELD champions from care homes.

**Aims**:

- **Day 1:** Understanding what PCC is and how homes can apply it in the care of residents. Developing ways to share this information with colleagues within the home.
- **Day 2:** Writing strengths-based care plans and providing tailored, structured social activities that recognise people’s abilities and interests, with the aim of providing 60 minutes per week per person.
- **Day 3:** Understanding the evidence about the use of antipsychotic medication and familiarisation with best practice guidelines and considering ways care homes could work with their local general practitioners.
- **Day 4:** Developing ways to understand the individual needs of people who are distressed (sometimes referred to as “challenging behaviour”) using formulation of a need-based model and identifying ways of using information gained through PCC/care planning (sessions 1 and 2) to meet these needs.

**Delivery style:** All sessions were manualised and involved didactic sessions, experiential learning, individual goal setting for each care home for dissemination of training information, and implementation activities between training sessions.

**(2) Months 6–9:** On-site consultation sessions totalling 8 hours per month with each care home, delivered flexibly, by negotiation, to best support each care home’s needs.

**(3) Concurrently in months 2–9:** Cascade training and implementation of activities.

**Training delivered by:** WHELD champions.

**Activities developed by:** WHELD champions and staff team members.

**Delivery style:** Adapted for care setting involved as standalone training sessions, modelling skills, incorporating sessions into daily routine, working with individual residents to develop personalised and tailored activities for 60 minutes a week, care home team formulation, and medication review and goal planning sessions to influence care planning.

### Outcome measures

All care home residents were assessed for dementia severity at baseline using the CDR [26], a validated scale that quantifies the severity of dementia using a structured interview, and the Functional Assessment Staging Tool (FAST) [27], a validated functional ordinal assessment scale for elderly people with dementia.

All outcome measures were assessed prior to randomisation and after 9 months of the intervention by a trained research assistant. The assessments at follow-up were collected by research assistants who had not previously visited the participating care homes. The research assistants were blind to treatment allocation, and every effort was made to maintain the blind by minimising contact between the research assistants and research therapists, ensuring that WHELD champions were not informants, and giving clear instructions to care homes and the research team to not disclose treatment allocation. The primary outcome was QoL, measured by the DEMQOL-Proxy [28], a 31-item interviewer-administered questionnaire answered by a caregiver, with a score range of 31 to 124 that assesses the QoL for people with dementia.

The secondary outcome measures included agitation assessed using the Cohen-Mansfield Agitation Inventory (CMAI) [29], a caregiver questionnaire of agitation completed through an interview with the caregiver, consisting of 29 items, each of which is rated on a 7-point scale of frequency. Information regarding antipsychotic use and the use of other psychotropic medications was recorded from medication charts. Overall neuropsychiatric symptoms were assessed with the Neuropsychiatric Inventory–Nursing Home Version (NPI-NH). The NPI-NH [30,31] was developed to assess psychopathology in patients with dementia in nursing homes and evaluates 12 neuropsychiatric disturbances common in dementia: delusions, hallucinations, agitation, dysphoria, anxiety, apathy, irritability, euphoria, disinhibition, aberrant motor behaviour, night-time behaviour disturbances, and appetite and eating abnormalities. The score of each item, if present, represents the product of symptom frequency and severity, with a maximum score of 12 for each domain. Secondary outcomes also included global deterioration (CDR) [26], mood (Cornell Scale for Depression in Dementia [CSSD]) [32], antipsychotic use, unmet needs (Camberwell Assessment of Need for the Elderly [CANE]) [33], quality of interactions (Quality of Interactions Scale [QUIS]) [34], pain (Abbey Pain Scale) [35], mortality, and cost.

Economic data for each individual in the study were collected using an adapted version of the Client Service Receipt Inventory (CSRI) [36,37], which includes questions about the individual’s sociodemographic profile, care home charges, and use of health and social care services. In addition, the staffing inputs of the optimised intervention were measured and covered time spent by the WHELD therapist and WHELD champion in training, supervision, and preparation.

### Randomisation and blinding

Nursing homes were allocated to receive either the WHELD intervention or TAU using secure web access to the remote randomisation centre at the North Wales Organisation for Randomised Trials in Health Clinical Trial Unit (NWORTH CTU) at Bangor University. Randomisation was performed by dynamic allocation [38] to protect against subversion while ensuring that the trial maintained a good balance to the allocation ratio of 1:1 both within each stratification variable and across the trial. Nursing homes were stratified by region and size. The system was coded and validated in the R statistical package. The NWORTH CTU generated randomisation codes and assigned clusters to intervention groups. This information was passed to the trial manager, and passed on to the principal investigator for each site only once all baseline evaluations had been completed.

Individual participants were consented and evaluated for dementia prior to randomisation of the nursing homes to minimise bias. Written consent was provided by participants when they had mental capacity to provide consent for their own participation. Written consent was provided by next of kin when individuals did not have mental capacity to consent for themselves. Clinicians and research assistants completing follow-up assessments were blind to treatment allocation. Every attempt was made to minimise accidental un-blinding by minimising contact between therapists and the researchers collecting outcome data and with clear instructions to researchers and nursing home staff to not discuss treatment allocation.

### Sample size

The target minimum sample size was 640 at the 9-month time point. Previous studies indicated that intra-home correlation coefficients rarely exceed 0.05. Taking this into account, a sample size of 640 participants therefore gives 90% power using a significance level of 5% to detect a standardised effect size of 0.3 SDs, which is generally accepted as the lowest threshold of a clinically meaningful benefit. The recruitment of a minimum of 840 participants allowed for loss of 200 through mortality or withdrawal, an important consideration in the context of the high morbidity and mortality of this group.

### Data analysis

Outcome measures for the study were assessed at baseline and at 9 months. All the outcome measures collected were described and reported using appropriate descriptive statistics and tabular and graphical techniques. Means with 95% confidence intervals are quoted, and a 5% significance level is reported. The Consolidated Standards of Reporting Trials (CONSORT) diagram information is presented in order to identify any differential dropout between the arms of the trial. The analysis of the quantitative outcomes was undertaken using a multilevel analysis of covariance (ANCOVA).

The primary outcome measure (DEMQOL-Proxy) and the secondary outcome measures were analysed using the multilevel modelling approach to ANCOVA, with the value at 9 months as the response. The baseline value was the covariate. The key factor was group (treatment [WHELD] or control [TAU]). The multilevel nature of the design was represented by 2 levels: care home and individual residents in the care home. Other covariates were number of residents in each cluster and the age, sex, and severity of dementia (FAST stage—baseline and follow-up) of participants with dementia. The provisional analysis plan was developed based on the analysis model developed for a previous smaller factorial study of the WHELD intervention [9]. In addition to the standard ANCOVA model, this included work to model and identify the best fit for the inclusion of baseline covariates and the evaluation of several imputation models. The same baseline covariate model was used in the final analysis plan for the current study. The imputation model was less predictive in validation analyses than it had been in the factorial study. The completer analysis was therefore used as the primary outcome in place of the imputation analysis. Therefore, the primary analysis included all participants with data available at the 9-month assessment point, and the imputation model was used as a sensitivity analysis. The analysis model was finalised prior to the locking of the study database for the current trial.

The same approach was used for the analysis of all secondary outcomes other than mortality, antipsychotic use, QUIS, and cost, except that the respective baseline variables were used as covariates rather than baseline DEMQOL-Proxy.

Mortality and antipsychotic use were compared between treatment groups using relative risk with 95% CI. QUIS used care-home-level data, and was compared between treatment groups using ANCOVA, but because of the smaller sample size did not use baseline covariates.

Further exploratory sub-group analysis was undertaken evaluating differences between WHELD and TAU in people with mild to moderate (FAST 4–5), moderately severe (FAST 6), and severe dementia (FAST 7) based on the recommendations of reviewers as part of the journal submission process. Based on reviewer recommendations, effect sizes and number needed to treat were also evaluated.

### Cost analysis

Total costs for each participant were derived from the collection of service use data for the 3-month period prior to the intervention (baseline) and the 9 months of the intervention (follow-up) and consisted of 3 main cost categories: intervention costs, accommodation charges, and health and social care costs. Intervention costs were calculated by deriving average hourly costs for WHELD champions and therapists, combined with time spent by each staff type on training, supervision, and intervention delivery, and were defined as a per participant cost. An additional cost was defined for the antipsychotic review element of the intervention for participants receiving antipsychotics. Accommodation costs were collected as weekly charges for each nursing home. Where this was unavailable or not known, the typical charge for a resident with a level of need similar to that of the participant in the study was obtained. Total health and social care costs consisted of services that are the main contributors to the cost of care in nursing homes: hospital inpatient, outpatient, day hospital, accident and emergency, primary care (calculated as per minute unit costs for general practitioners and practice nurses), community health care, and ambulatory care. Data on each nursing home resident’s use of health care (obtained from the CSRI) were multiplied by appropriate unit costs to calculate health and social care costs for each participant at each time point. Mean differences in costs and 95% CIs were obtained by non-parametric bootstrapped regression (1,000 repetitions) modelling to account for non-normal distributions. A multilevel mixed model was used, controlling for site and age at entry into the study. The adjusted total health and social care cost and outcome models also included the treatment variable as a random effect at the care home level. Clustering was accounted for by allowing the model intercept and treatment variable coefficient (i.e., treatment effect) to vary by care home.

### Sensitivity analysis

As a sensitivity analysis, the same analysis was undertaken for the primary and key secondary outcomes but using imputed values for people who did not complete the 9-month follow-up. Logistic regression was used to predict missing variables from the factors and covariates measured at baseline, using the approach validated in a previous factorial study [9].

Data are deposited in the Dryad Digital Repository (doi: 10.5061/dryad.75373) [39].

## Results

### Cohort characteristics

**Fig 1:**
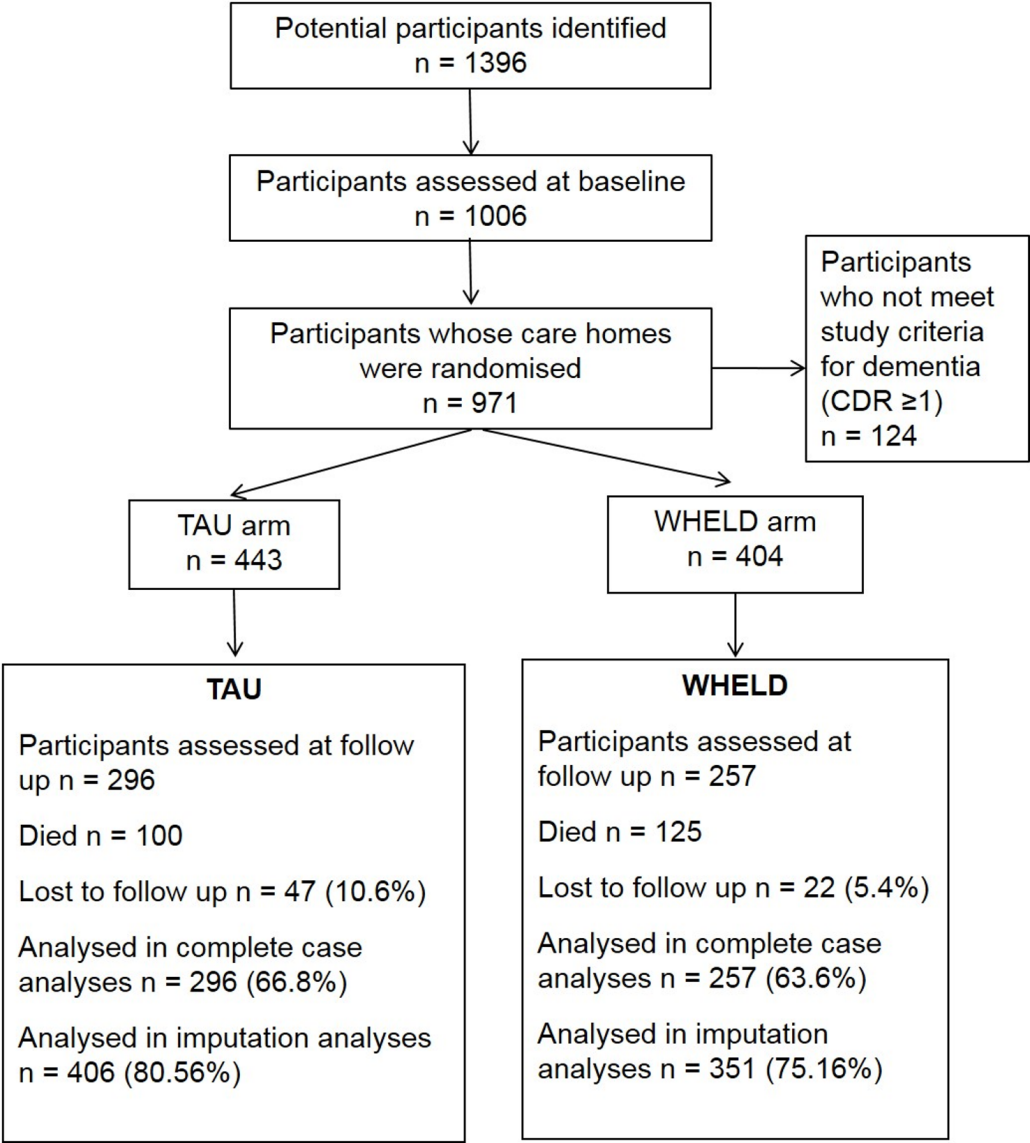
CONSORT chart showing flow of participants through the study. CDR, Clinical Dementia Rating; TAU, treatment as usual.

In all, 1,006 participants were consented to the study, with 847 individuals randomised to TAU or WHELD. The majority of participants had moderately severe or severe dementia, and 71% were female. Follow-up assessments were available for 553 participants. Mortality accounted for the majority of participants who did not complete follow-up assessments. The descriptive statistics for participants who completed the follow-up were similar to those for the original population, although numerically marginally more residents receiving TAU completed the follow-up compared to those receiving the WHELD intervention (66.8% versus 63.6%). The baseline characteristics of the study participants are described in Table 1, and flow of participants through the study is presented in Fig 1. The trial ended after the last follow-up assessment of the last participant was completed.

**Table 1:** Descriptive statistics for baseline cohort and completers.

Characteristic	Baseline cohort (*n* = 847)		Completers (*n* = 553)	
	TAU	WHELD	TAU	WHELD
**Total population**	**443 (100%)**	**404 (100%)**	**296 (66.82%)**	**257 (63.61%)**
**Sex**				
Male	129 (29.1%)	132 (32.7%)	84 (28.4%)	78 (30.4%)
Female	314 (70.9%)	272 (67.3%)	212 (71.6%)	179 (69.6%)
**Age (years)**	**88.5 (0.50)**	**88.4 (0.57)**	**86.6 (0.50)**	**86.6 (0.53)**
**FAST stage**				
Mild dementia or less	35 (7.90%)	47 (11.64%)	21 (7.09%)	23 (8.95%)
Moderate dementia	38 (8.58%)	39 (9.65%)	15 (5.07%)	16 (6.22%)
Moderately severe dementia	267 (60.27%)	241 (59.65%)	159 (53.71%)	153 (59.53%)
Severe dementia	103 (23.23%)	77 (19.06%)	101 (34.12%)	65 (25.29%)
**Antipsychotic use**	78 (9.2%)	75 (8.9%)	51 (9.2%)	52 (9.4%)
**DEMQOL-Proxy score**	103.84 (0.70)	103.04 (0.74)	103.69 (0.68)	105.62 (0.59)
**CMAI**	48.49 (1.03)	48.29 (1.04)	48.10 (1.06)	46.00 (1.01)
**NPI-NH**	2.13 (0.13)	2.36 (0.23)	2.14 (0.14)	2.33 (0.24)

Data are given as *n* (percent) or mean (SEM).

CMAI, Cohen-Mansfield Agitation Inventory; FAST, Functional Assessment Staging Tool; NPI-NH, Neuropsychiatric Inventory–Nursing Home Version; TAU, treatment as usual.

### Outcome measures

The WHELD intervention conferred a statistically significant 2.54-point (SEM 0.88) improvement in QoL compared to TAU (95% CI 0.81, 4.28; Cohen’s *D* 0.24) as measured by the DEMQOL-Proxy over 9 months (*Z* score 2.82, *p =* 0.0042; mean difference 2.54, SEM 0.88). On the secondary outcomes, WHELD also conferred a statistically significant 4.27-point (*Z* score 2.68, *p =* 0.0076; mean difference 4.27, SEM 1.59; 95% CI −7.39, −1.15; Cohen’s *D* 0.23) benefit on the CMAI compared to TAU with respect to agitation and conferred a statistically significant 4.55-point (*Z* score 3.52, *p* < 0.001; mean difference 4.55, SEM 1.28; 95% CI −7.07,−2.02; Cohen’s *D* 0.30) benefit on the total NPI-NH compared to TAU with respect to overall neuropsychiatric symptoms. The multilevel mixed-effects linear or logistic regression models for primary and secondary outcome measures are shown in Table 2.

**Table 2:** Effect estimates of WHELD intervention in comparison to TAU on primary outcome and key secondary outcome measures (multiple imputation analysis).

Outcome measure	Adjusted effect (SE)*	*p*-Value	Mean difference (SEM)	95% CI of mean difference	Effect size (Cohen’s *D*)	Number needed to treat^∆^
DEMQOL-Proxy (*n =* 553)	*R =* 0.12 *Z =* 2.82	0.0042	2.54^+^ (0.88)	0.81, 4.28	0.24	9
CMAI (*n =* 553)	*R* = 0.11; *Z* = 2.68	0.0076	4.27^+^ (1.59)	−7.39, −1.15	0.23	6
NPI-NH (*n =* 547)	*R =* −1.5; *Z =* 3.52	<0.001	4.55^+^ (1.28)	−7.07, −2.02	0.30	9

*Adjusted effect takes into account baseline value, age, sex, Clinical Dementia Rating, site, and clustering within care homes.

^∆^Based on binary outcome: better than mean overall outcome versus mean outcome or worse than overall mean outcome for DEMQOL and CMAI.

^+^DEMQOL: improvement in WHELD group from baseline to 9 months 4.78, improvement in TAU group 2.24, mean difference 2.54. CMAI: improvement in WHELD group from baseline to 9 months −4.13, worsening in TAU group 0.14, mean difference 4.27. NPI-NH: improvement in WHELD group from baseline to 9 months −2.64, worsening in TAU group 1.91, mean difference 4.55.

CMAI, Cohen-Mansfield Agitation Inventory; NPI-NH, Neuropsychiatric Inventory–Nursing Home Version; TAU, treatment as usual.

Prescriptions of antipsychotic medications were stable across the study in both treatment groups, with no reduction in antipsychotic use in the WHELD treatment group compared to TAU (change in antipsychotic use: WHELD −0.1%, SEM 0.1; TAU −0.2%, SEM 0.1, *p =* 0.60; antipsychotic use at 9 months: WHELD versus TAU relative risk 1.06, 95% CI 0.62 to 1.82, *p =* 0.82). Regarding other secondary outcomes, there were no statistically significant differences between the WHELD and TAU groups for change in global deterioration (CDR *Z* score 0.053, *p =* 0.96; mean difference 0.01, SEM 0.22), unmet needs (CANE *Z* score 0.84, *p =* 0.62; mean difference 0.04, SEM 0.08), pain (Abbey Pain Scale *Z* score 1.084, *p =* 0.27; mean difference 0.33, SEM 0.31), or mood (CSSD *Z* score 0.036, *p =* 0.97; mean difference 0.02, SEM 0.48). There were no significant interaction effects in the primary analysis model, and further analyses accounting for interactions were therefore not undertaken.

The quality of interactions of positive care between care staff and residents with dementia (QUIS) was collected as a care-home-level assessment in 62 of the participating care homes. There was a statistically significant 19.7% greater increase in the proportion of positive care interactions from baseline to 9 months in the WHELD group compared to the TAU group (SEM 8.94; 95% CI 2.12, 37.16, *p =* 0.03; Cohen’s *D* 0.55).

A sub-group analysis was also undertaken comparing the WHELD intervention in people with mild to moderate (FAST 4–5), moderately severe (FAST 6), and severe (FAST 7) dementia, focusing on the primary and key secondary outcomes (DEMQOL, CMAI, and NPI-NH). Statistically significant benefits of similar magnitude to the benefits in the overall population were seen in people with moderately severe dementia for QoL, agitation, and overall neuropsychiatric symptoms, but there were no statistically significant benefits on QoL in the smaller groups of individuals with mild to moderate or severe dementia. The full results are shown in Table 3.

**Table 3:** Effect estimates of the WHELD intervention in comparison to treatment as usual for key outcome measures (multiple imputation analysis): Sub-analysis evaluating impact of WHELD in mild to moderate, moderately severe, and severe dementia.

Outcome	Adjusted effect (SE)*	*p*-Value	Mean difference (SEM)	95% confidence interval of mean difference
**DEMQOL-Proxy**				
Severe dementia	*R =* 0.00; *Z =* 0.03	0.97	−0.06 (1.72)	−3.43, 3.32
Moderately severe dementia	*R =* 0.20; *Z =* 3.62	<0.001	4.28 (1.16)	2.01, 6.56
Mild to moderate dementia	*R =* 0.06; *Z =* 0.61	0.54	1.11 (1.83)	−2.47, 4.69
**CMAI**				
Severe dementia	*R =* −0.06; *Z =* 0.55	0.58	−2.24 (4.05)	−10.17, 5.69
Moderately severe dementia	*R =* −0.12; *Z =* 2.08	0.04	−4.52 (2.17)	−8.77, −0.27
Mild to moderate dementia	*R =* −0.18; *Z =* 1.93	0.05	−4.57 (2.34)	−9.15, 0.01
**NPI-NH**				
Severe dementia	*R =* 0.19; *Z =* 1.91	0.05	−5.73 (2.90)	−11.42, −0.04
Moderately severe dementia	*R =* 0.15; *Z =* 2.74	0.006	−4.83 (1.75)	−8.26, −1.39
Mild to moderate dementia	*R =* 0.14; *Z =* 1.54	0.13	−3.05 (1.99)	−6.94, 0.84

*Adjusted effect takes into account baseline value, age, sex, Clinical Dementia Rating, site, and clustering within care homes.

CMAI, Cohen-Mansfield Agitation Inventory; NPI-NH, Neuropsychiatric Inventory–Nursing Home Version.

The sensitivity analysis using imputed values confirmed that WHELD conferred a statistically significant benefit in DEMQOL-Proxy (mean difference 0.06, 1.5 SEM; *Z* score 2.50, *p =* 0.015), CMAI (mean difference 0.08, 1.96 SEM; *Z* score 2.06, *p =* 0.04), and NPI-NH (mean difference −2.45, SEM 0.066; *Z* score 2.64, *p =* 0.01) outcomes compared to TAU.

### Adverse events

A total of 549 serious adverse events were recorded during the period of the trial. The events were balanced between the 2 treatment groups, with no statistical differences (291 events in the WHELD group and 258 in the TAU group; Table 4). There was no significant difference in mortality between the WHELD and TAU group (relative risk 1.08, 95% CI 0.86 to 1.35, *p =* 0.50).

**Table 4:** Serious adverse event reporting by category and WHELD treatment group.

SAE category	Group		Total
	WHELD	TAU	
Dehydration	8	2	10
Fall	30	14	44
Fractures	15	13	28
Mortality	122	103	225
Pneumonia	16	12	28
Stroke	3	8	11
Delirium	0	1	1
Chest infections	26	15	41
Renal	2	1	3
Increased confusion	4	0	4
UTI	11	7	18
Pulmonary embolism	1	0	1
Other	53	82	135
**Total per group**	291	258	549

SAE, serious adverse event; TAU, treatment as usual; UTI, urinary tract infection.

### Cost analysis

The direct cost of delivering the intervention compared to TAU was £8,627 more per home. Fifty-three percent (£4,554) of the cost related to WHELD champion time spent in training and supervision. The remaining costs related to therapist time. Delivery of the intervention to residents incurred an additional £130 per person per month. The additional cost incurred for antipsychotic review was £23 per resident, which accounted for WHELD champion time spent reviewing antipsychotic use in 16% of residents and contacting prescribing physicians. Analysis of service use showed higher healthcare costs unrelated to the intervention in the TAU group compared to the WHELD intervention group. Participants receiving the intervention showed a significant health and social care cost advantage. Taking into account the cost of the intervention and the total health and social care costs, there was a cost advantage for the WHELD treatment (Table 5).

**Table 5:** Unadjusted mean costs and mean cost differences at baseline and over 9 months (British pounds, 2014–2015).

Cost category	Intervention		TAU		Intervention versus TAU*	
	Mean (£)	SD (£)	Mean (£)	SD (£)	Unadjusted mean difference (£)	95% CI
**WHELD intervention**	2,713	121	0	—	2,713	2,701 to 2,724
**Baseline (*n =* 887)**						
Accommodation charges	9,480	2,010	10,233	3,675	−753	−1,128 to −365
Hospital	387	1,759	407	2,413	−20	283 to 242
Primary care	96	126	98	148	−2	19 to 14
Community health	23	80	19	79	4	−7 to 14
Emergency	12	37	9	34	3	−1 to 7
Total health and social care costs	9,998	2,601	10,766	4,396	−768	−1,249 to −338
**9-month follow-up (*n =* 553)**						
Accommodation charges	28,606	10,863	33,005	12,428	−4,399	−5,725 to −2,898
Hospital	269	1,166	262	1,267	7	−183 to 188
Primary care	700	294	1,020	301	−320	−364 to −277
Community health	78	260	70	206	8	−23 to 44
Emergency	49	133	85	244	−36	−68 to −10
Total health and social care costs	29,702	8,774	34,442	11,106	−4,740	−6,129 to −3,156

*Cost comparisons for 9 months include covariates for site, age, baseline CMAI score, and baseline value of the same cost variable. Baseline cost comparisons include covariates for site, age, and baseline CMAI score.

CMAI, Cohen-Mansfield Agitation Inventory; TAU, treatment as usual.

## Discussion

In what is, to our knowledge, the largest RCT conducted of a staff training and non-pharmacological intervention for people with dementia living in nursing homes, we have demonstrated that the WHELD intervention confers a statistically significant improvement in QoL over 9 months. There was also a statistically significant benefit regarding agitation and overall neuropsychiatric symptoms over the 9-month period. Whilst the effect sizes were small, the benefits in agitation and neuropsychiatric symptoms were comparable to (agitation) or better than (NPI-NH) the benefits seen with antipsychotic drugs. There was also a significant increase in the proportion of positive care interactions between care staff and residents with dementia, with a moderate effect size. Importantly, the benefits were achieved in the context of a cost saving and used a model that can readily be implemented in nursing homes. Antipsychotic drug use was stable in both treatment groups across the study, and the WHELD treatment intervention did not reduce antipsychotic use (baseline frequency was very low).

Despite the importance of QoL, few trials have examined the impact of interventions on this outcome. In this study, the WHELD intervention conferred a statistically significant improvement in QoL over 9 months, building on a previous proof-of-concept study of WHELD. That study reported a reduction in QoL following antipsychotic review that was mediated by social interaction within the context of an overall PCC training paradigm for care staff [9]. We would speculate that the added benefit was probably a reflection of the structured approach to promoting pleasant activities involving social interaction. The subsequent optimisation of the WHELD intervention maintained benefit but reduced overall cost, making it a cost-effective programme for delivery in care homes. A sub-group analysis focusing on people with mild to moderate, moderately severe, and severe dementia indicated that benefits on QoL were more robust in people with moderately severe dementia.

Agitation is a frequent and distressing symptom for people with dementia living in nursing homes [10,11,40]. Benefits from pharmacological treatment with atypical antipsychotics are limited to modest improvements in aggression [12,13,15], and the significant advantage on the CMAI for the WHELD intervention compared to control is comparable to the modest treatment advantage for atypical antipsychotics from a meta-analysis of previous RCTs [12,13,15]. In addition, the use of atypical antipsychotics is limited by the significant adverse effects of these medications in people with dementia [12,13,15]. Recent studies have begun to suggest that other pharmacological therapies such as citalopram [20] and dextromethorphan [21] may confer significant benefit for the treatment of agitation, but further studies are needed. There was also a statistically significant benefit in overall neuropsychiatric symptoms conferred by the WHELD intervention compared to TAU, suggesting a breadth of benefit beyond just reducing agitation.

Although comparable to the effect sizes of atypical antipsychotics, the standardised effect sizes of benefit for WHELD were small in the context of a clinical intervention. The benefits did however include benefits in QoL, which have not been demonstrated with pharmacological interventions. Although there is no established threshold for a clinically meaningful benefit in QoL, any statistically significant benefit is important given the absence of any benefit in previous studies. In addition, the intervention was not just delivered to people with clinically significant neuropsychiatric symptoms, but conferred benefit amongst a broader population of people with dementia living in care homes. Whilst the effect sizes would be considered marginal in terms of a clinically significant benefit, we believe that the benefits to the broader population of people with dementia in care homes make this a meaningful benefit in the quality of care.

This study is consistent with the evidence base but provides important and novel data within the literature. Our results also compare favourably to those of the small number of published intervention studies that have focused on promoting PCC, none of which have reported benefits in QoL [6,7]. The findings are particularly favourable when compared with those of trials of antipsychotic medications, which show only very modest benefits over 12 weeks in the context of significant harms [41,42]. In addition, the current study shows cost advantages over usual care, which has not been demonstrated, to our knowledge, with any previous drug or non-drug intervention.

Elements of the WHELD intervention, such as social interaction and pleasant events, have previously been demonstrated to improve agitation in modest-sized RCTs [22,23]. Incorporating them within a coherent framework such as WHELD enables straightforward and affordable implementation of these approaches in clinical and care practice.

Interestingly, there was a low baseline use of antipsychotic medications (<10%) in this study, reflecting the major changes in clinical practice and the reductions in antipsychotic use that have been achieved for people with dementia in the last decade. In contrast to our previous factorial RCT of the WHELD intervention, no significant reduction in antipsychotic use was achieved, and antipsychotic use was stable in both groups. This is likely attributable to a combination of the low baseline levels of antipsychotic prescription and the more limited education programme for primary care physicians within the current study than in the previous factorial RCT, and highlights the potential additional value of primary care education programmes in parallel to care home training.

This study was a robust, well-powered RCT evaluating the sustained impact of combining PCC and evidence-based non-pharmacological interventions for people with dementia in nursing homes. The study had good retention of surviving participants compared to most studies conducted in nursing home settings. The intervention evaluated was pragmatic, fully manualized, and designed so that it can be easily disseminated and implemented in routine clinical practice. There were also limitations. Antipsychotic review was based on augmenting processes within care homes to trigger medical review and did not in this study involve proactive primary care education. In addition, although the study used a well-validated method, evaluating QoL in people with dementia is challenging and all methods have some limitations. High mortality rates are usual in studies of frail groups of individuals living in care homes, but lead to high non-completion rates and present some challenges for data analysis and interpretation. One of the original secondary outcomes was to evaluate the impact of the intervention on the care home environment. The selected scale focused mainly on the physical building rather than other aspects of the environment, and the programme management group therefore decided to omit this measure at 9-month follow-up as it was unlikely that substantial building renovations had taken place in any of the participating care homes.

A key issue for future studies is the sustainability of the intervention, particularly with turnover of staff, including the WHELD champions. To be sustainable, the WHELD intervention needs to be firmly embedded within the care home culture, and it will be important for further research to identify the optimal approach to maintain benefits. As WHELD is largely verbally based, it will also be important to further evolve interventions more tailored to the needs of people with more severe dementia.

## Supporting information

S1 Table Summary and examples of WHELD training. (DOCX)

S1 Text CONSORT chart showing flow of participants through the 9-month study. In all, 847 participants were randomised, of whom 757 were included in the full imputation analysis.
(PDF)

S2 Text Data analysis protocol for the RCT, as developed for this study and the associated factorial study [25]. (DOCX)

## Abbreviations

ANCOVA: analysis of covariance
CANE: Camberwell Assessment of Need for the Elderly
CDR: Clinical Dementia Rating
CMAI: Cohen-Mansfield Agitation Inventory
CSRI: Client Service Receipt Inventory
CSSD: Cornell Scale for Depression in Dementia
FAST: Functional Assessment Staging Tool
NPI-NH: Neuropsychiatric Inventory–Nursing Home Version
NWORTH CTU: North Wales Organisation for Randomised Trials in Health Clinical Trial Unit
PCC: person-centred care
QoL: quality of life
QUIS: Quality of Interactions Scale
RCT: randomised controlled trial
TAU: treatment as usual
