# Supplementary material for: Impact of person-centred care training and person-centred activities on quality of life, agitation, and antipsychotic use in people with dementia living in nursing homes: A cluster-randomised controlled trial
Source: PLoS Med. 2018 Feb 6;15(2):e1002500. doi: 10.1371/journal.pmed.1002500 (PMC5800565; doi:10.1371/journal.pmed.1002500)
Supplement: S1 Table — (DOCX) [file pmed.1002500.s001.docx]

**Supplementary Table 1**

**Summary and Examples of WHELD Training Topics**

| **Training Focus** | **Training for Dementia Champions** | **Dementia Champions cascade to care home colleagues** | **Examples of practice developed in care homes** |
| --- | --- | --- | --- |
| **Person Centred Care** | Understanding individuals and their experience of dementia.  Completing life stories  Improving communication skills with people with cognitive or sensory impairments. | Experiential simulation sessions of having sensory deficits and receiving care.  Created a “one page profile” of what’s important to an individual to use as the front page of care plan.  Modelling ways to approach people, matching verbal and non-verbal skills, coaching colleagues to share good practice. | Slowing the pace of dressing with a man who was resistant to personal care.  A woman started being taken to the swimming baths in recognition of her long- standing interest and skill  Staff facilitated social topics of conversation ( based on life story) not just “task based talk”  Staff checked that people were wearing glasses and hearing aids if needed. |
| **Social Intervention** | Using strengths based language for care planning  Identifying positive social preferences  Goal planning for individuals  Running groups and adapting individual activities. | Critiquing care plans with colleagues to identify resident’s skills.  Discussing life story, current observed preferences and involving resident and family to identify pleasant social activities  Developing SMART goal planning and setting review times  Matching resident activity preferences with staff activity preferences  Creation of an “activity toolbox” with a range of items of interest, which could be used spontaneously for social activity and conversation for periods of 5-10 minutes. | One home develop a “guide to words and phrases” that promote a positive approach.  Staff recognized that a man who was known to “like music” was a jazz fan and tailored the music and conversation to this.  Staff developed a plan to increase the amount a woman was able to walk to a personally meaningful destination.  Many teams created an “Interests chart” for the staff room to easily see skills and interest of residents and staff.  Staff interested in gardening set up a group with residents to plant seeds.  Puzzles, postcards, books, music, fabric was used to stimulate conversation and activity with staff and visitors. |
| **Review of Antipsychotic Medication** | National guidelines on antipsychotic prescribing  Ways of identifying and recording BPSD and possible medication side effects.  Planning how to work together with local GPs | Sharing information with key colleagues to increase awareness of potential risks.  Working with colleagues to adapt existing processes and records to increase clarity  Proactively talking with doctors about the best way to have 12 week reviews. | Care plans specifying a 12 week review date were developed  Abbey Pain Scale was used to assess resident  A log of residents’ prescribed antipsychotic medication was reviewed at every GP visit. |
| **Understanding needs and behaviour** | Recognising changes in behaviour as an expression of needs  Biopsychosocial factors which contribute to needs  Understanding triggers to changes in behaviour.  Communication cycles between staff and residents and how they influence relationships. | Simulation sessions with colleagues to identify frustrations residents may experience.  Structured team meetings to share knowledge about residents and agree an understanding of their needs  Using ABC charts and monitoring forms  Reflective sessions for staff on their perceptions and interactions | Homes made changes to their environment to reduce noise and “clutter”.  Families were consulted about residents’ past routines and coping styles  A resident who was thought to “be bored” was invited to help with housekeeping tasks which they enjoyed.  Ways of monitoring were incorporated into routine planning.  Early signs of distress were recognised and responded to.  Staff tried different ways of interacting and reflected on the effect. |
