# Supplementary material for: Impact of person-centred care training and person-centred activities on quality of life, agitation, and antipsychotic use in people with dementia living in nursing homes: A cluster-randomised controlled trial
Source: PLoS Med. 2018 Feb 6;15(2):e1002500. doi: 10.1371/journal.pmed.1002500 (PMC5800565; doi:10.1371/journal.pmed.1002500)
Supplement: S2 Text — (DOCX) [file pmed.1002500.s003.docx]

**Data Analysis Section Submitted Paper**

Outcome measures for the study were assessed at baseline and at nine months. All the outcome measures collected were described and reported using appropriate descriptive statistics and tabular and graphical techniques. Means with 95% confidence intervals were quoted and a 5% significance level was reported. The Consolidated Standards of Reporting Trials (CONSORT) diagram information is presented in order to identify any differential dropout between the arms of the trial. The analysis of the quantitative outcomes was undertaken using a multilevel analysis of covariance (ANCOVA).

The primary outcome measure (DEMQOL proxy) and the secondary outcome measures were analysed using the multilevel modelling approach to ANCOVA, with the value at nine months as the response. The baseline value was the covariate. The key factor was group (treatment (WHELD or control –Treatment as Usual). The multilevel nature of the design was represented by two levels: care home and individual residents in the care home. Other covariates were number of residents in each cluster and the age, gender and severity of dementia (FAST stage –baseline and follow-up) of participants with dementia. The provisional analysis plan was developed based on the analysis model developed for our previous smaller factorial study of the WHELD intervention (9). In addition to the standard ANCOVA model, this included work to model and identify the best model for the inclusion of baseline co-variates and evaluation of several imputation models. The same baseline covariate model was used in the final analysis plan for the current study. The imputation model was less predictive in validation analyses than it had been in the factorial study. The completer analysis was therefore analysed as the primary outcome in place of the imputation analysis. Therefore the primary analysis included all participants with data available at the nine month assessment point, and the imputation model was used as a sensitivity analyses. The analysis model was finalized prior to the locking of the study database for the current trial.

The same approach was used for the analysis of all secondary outcomes other than mortality , antipsychotic use, QUIS and cost. Mortality and antipsychotic use were compared between treatment groups using Relative Risk with 95% CI. QUIS used care home level data, and was compared between treatment groups using ANCOVA, but because of the samller sample size did not use baseline covariates.

Further exploratory sub-group analysis were undertaken evaluating differences between WHELD and treatment as usual in people with mild-moderate, moderately severe and severe dementia respectively based on the recommendations of the reviewers as part of the journal submission process. Based on these recommendations effect sizes and Number Needed to Treat were also evaluated.

**From Whitaker et al Trials 2014**

Outcome measures for the study will be assessed at baseline and at nine months. All the outcome measures collected will be described and reported using appropriate descriptive statistics and tabular and graphical techniques. Means with 95% confidence intervals will be quoted and a 5% significance level will be reported. The Consolidated Standards of Reporting Trials (CONSORT) diagram information will be presented in order to identify any differential dropout between the arms of the trial. The analysis of the quantitative outcomes will involve a multilevel analysis of covariance (ANCOVA). Logistic regression will be used to assess whether the missing status can be predicted from any of the factors and covariates measured at baseline. The variables which are identified to affect the missing status will be included in the analysis model [31]. The primary outcome measure (DEMQOL proxy) [22] and the secondary outcome measures will be analysed using the multilevel modelling approach to ANCOVA. The value at nine months will be the response. The baseline value will be the covariate. The key factor will be group (treatment or control). The multilevel nature of the design will be represented by two levels: care home and individual residents in the care home. Other covariates, whether at the care home level (for example, number of residents) or the resident level (age, gender) will be investigated to see whether they have an effect on the response measure and included as necessary.

A full statistical analysis plan will be developed through the course of the study.
